# 3-(1-Adamantylamino)-3-methyl-1-phenyl­quinoline-2,4(1*H*,3*H*)-dione

**DOI:** 10.1107/S1600536809026464

**Published:** 2009-07-15

**Authors:** Niusha Mahmoodi, Marek Nečas, Robert Vícha

**Affiliations:** a Department of Chemistry, Faculty of Technology, Tomas Bata University in Zlin, Nám. T. G. Masaryka 275, Zlín,762 72, Czech Republic; b Department of Chemistry, Faculty of Science, Masaryk University in Brno, Kamenice 5, Brno-Bohunice, 625 00, Czech Republic

## Abstract

The structure of the title compound, C_26_H_28_N_2_O_2_, contains essentially planar quinoline and benzene rings, the maximum deviations from the best plane being 0.086 (2) and 0.0056 (19) Å, respectively; the dihedral angle between the rings is 82.87 (4)°. The adamantane cage consists of three fused cyclo­hexane rings in classical chair conformations, with C—C—C angles in the range 107.85 (15)–111.35 (15)°. Enanti­omers are linked alternately into chains along the *c* axis *via* short N—H⋯O inter­actions and further C–H⋯π inter­actions stabilize pairs of enanti­omers, forming a two-dimensional network.

## Related literature

For the synthesis and biological activity of related compounds, see: Kafka *et al.* (2002 ▶); Nayyar *et al.* (2007 ▶). For the properties of adamantane-containing compounds, see: van Bommel *et al.* (2001 ▶). For a related structure, see: Shishkina *et al.* (2001 ▶). For background to C—H⋯π inter­actions, see: Nishio (2004 ▶); Jorgensen & Severance (1990 ▶).
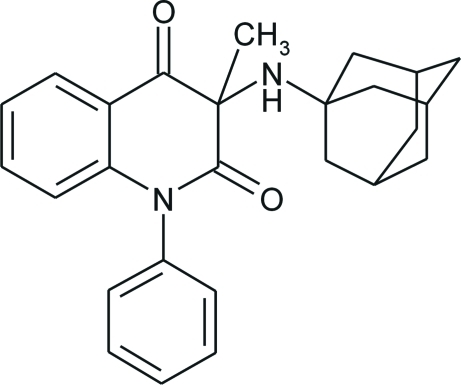


## Experimental

### 

#### Crystal data

C_26_H_28_N_2_O_2_
*M* *_r_* = 400.50
Monoclinic, 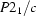

*a* = 9.9714 (4) Å
*b* = 24.1041 (11) Å
*c* = 9.3805 (5) Å
β = 113.111 (5)°
*V* = 2073.68 (17) Å^3^
*Z* = 4
Mo *K*α radiation
μ = 0.08 mm^−1^
*T* = 120 K
0.30 × 0.30 × 0.20 mm

#### Data collection

Kuma KM-4 CCD diffractometer
Absorption correction: none
22477 measured reflections
3648 independent reflections
2226 reflections with *I* > 2σ(*I*)
*R*_int_ = 0.051

#### Refinement

*R*[*F* ^2^ > 2σ(*F* ^2^)] = 0.041
*wR*(*F* ^2^) = 0.106
*S* = 0.88
3648 reflections
272 parameters
H-atom parameters constrained
Δρ_max_ = 0.53 e Å^−3^
Δρ_min_ = −0.25 e Å^−3^

### 

Data collection: *Xcalibur* (Oxford Diffraction, 2006 ▶); cell refinement: *Xcalibur*; data reduction: *Xcalibur*; program(s) used to solve structure: *SHELXS97* (Sheldrick, 2008 ▶); program(s) used to refine structure: *SHELXL97* (Sheldrick, 2008 ▶); molecular graphics: *ORTEP-3* (Farrugia, 1997 ▶) and *Mercury* (Macrae *et al.*, 2008 ▶); software used to prepare material for publication: *SHELXL97*.

## Supplementary Material

Crystal structure: contains datablocks global, I. DOI: 10.1107/S1600536809026464/pk2176sup1.cif

Structure factors: contains datablocks I. DOI: 10.1107/S1600536809026464/pk2176Isup2.hkl

Additional supplementary materials:  crystallographic information; 3D view; checkCIF report

## Figures and Tables

**Table 1:** Hydrogen-bond geometry (Å, °)

*D*—H⋯*A*	*D*—H	H⋯*A*	*D*⋯*A*	*D*—H⋯*A*
N1—H1*A*⋯O2^i^	0.88	2.29	3.125 (2)	158
C25—H25*A*⋯*Cg*1^ii^	0.95	2.91	3.659 (2)	136

Symmetry code: (i) 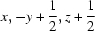; (ii) 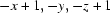. *Cg*1 is the centroid of the C13–C18 ring.

**Fig. 1.**
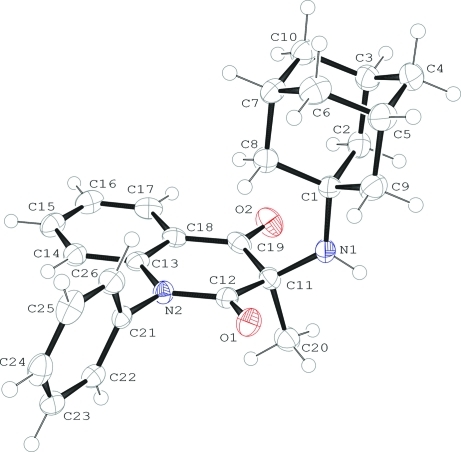
Ellipsoid plot of the asymmetric unit with atoms represented at 50% probability.

**Fig. 2.**
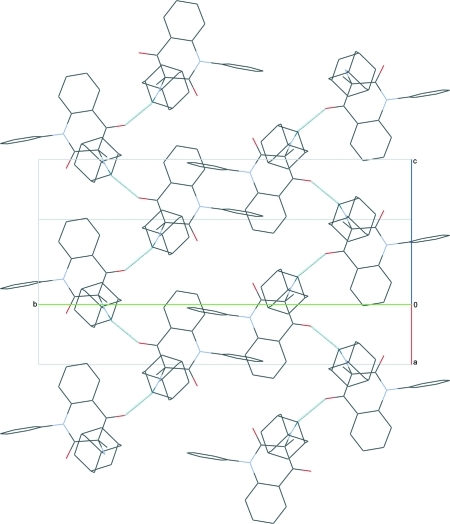
The crystal packing viewed perpendicular to the bc plane. Hydrogen atoms are omitted except for those participating in H-bonds.

## supplementary crystallographic information

### Comment

A number of compounds that include the quinoline moiety have well known chemotherapeutical properties. From a pharmacological point of view, two very important and seemingly contradictory properties may be improved when the adamantane substituent is introduced into biologically active compounds. The solubility in aqueous media may be enhanced by complexation of adamantane with β-cyclodextrin and the liphophilic adamantane cage may accelerate permeability through biological membranes (van Bommel *et al.*, 2001). Recently, some quinolines bearing adamantyl substituents have been introduced as promising anti-tuberculosis agents (Nayyar *et al.*, 2007).

The molecule of the title compound (Fig. 1) consists of planar benzene and quinoline rings with maximum deviations from the best plane being 0.0056 (18) Å for C23 and 0.086 (2) Å for C12, respectively. The dihedral angle between quinoline and benzene rings is 82.87 (4)°. The torsion angles describing the alignment of the adamantane and quinoline moiety C12–C11–N1–C1 and C11–N1–C1–C8 are -73.9 (2)° and 16.8 (2)°, respectively. Enantiomers alternate in chains along the *c* axis, and are linked *via* N1–H1a···O2 short interactions (Table 1, Fig. 2). Pairs of inverse enantiomers are stabilized by edge-to-face C–H···π interactions with the H···*C*g distance being 2.914 (2) Å (*C*g is the centroid of C13–C18).

### Experimental

The title compound was prepared according to a slightly modified literature procedure of Kafka *et al.* (2002). Adamantane-1-amine hydrochloride (200 mg, 1.07 mmol) was dissolved in 3 ml of DMF and triethylamine (212 mg, 2.1 mmol) was added dropwise at 273 K. Into this mixture, a solution of *N*-phenyl-3-chloro-3-methylquinoline-2,4-dione (153 mg, 0.535 mmol) in 3 ml of DMF was added dropwise at 273 K. The resulting solution was stirred for 93 h at room temperature until starting material disappeared (according to TLC).  The mixture was poured into crushed ice, extracted several times with diethyl ether, the combined organic portions were dried over sodium sulfate and the crude product was obtained after evaporation of solvent under reduced pressure.  The title compound was isolated from complex crude material by column chromatography (silica gel, ethyl acetate:hexane 1:4 *v*/*v*) as a pale yellow crystalline powder (53 mg, 25%, mp 449–451 K). The single crystal suitable for X-ray analysis was obtained by spontaneous evaporation from chloroform solution at 298 K.

### Refinement

Hydrogen atoms were positioned geometrically and refined as riding using standard *SHELXL-97* facilities, with their *U*_iso_ set to either 1.2*U*_eq_ or 1.5*U*_eq_(methyl) of their parent atoms.

### Crystal data

**Table:**

C_26_H_28_N_2_O_2_	*F*(000) = 856
*M**_r_* = 400.50	*D*_x_ = 1.283 Mg m^−^^3^
Monoclinic, *P*2_1_/*c*	Melting point = 451–449 K
Hall symbol: -P 2ybc	Mo *K*α radiation, λ = 0.71073 Å
*a* = 9.9714 (4) Å	Cell parameters from 24803 reflections
*b* = 24.1041 (11) Å	θ = 2.8–27.5°
*c* = 9.3805 (5) Å	µ = 0.08 mm^−^^1^
β = 113.111 (5)°	*T* = 120 K
*V* = 2073.68 (17) Å^3^	Block, yellow
*Z* = 4	0.30 × 0.30 × 0.20 mm

### Data collection

**Table:**

Kuma KM-4 CCD diffractometer	2226 reflections with *I* > 2σ(*I*)
Radiation source: fine-focus sealed tube	*R*_int_ = 0.051
graphite	θ_max_ = 25.0°, θ_min_ = 2.8°
Detector resolution: 0.06 pixels mm^-1^	*h* = −9→11
ω scans	*k* = −28→28
22477 measured reflections	*l* = −11→11
3648 independent reflections	

### Refinement

**Table:**

Refinement on *F*^2^	Primary atom site location: structure-invariant direct methods
Least-squares matrix: full	Secondary atom site location: difference Fourier map
*R*[*F*^2^ > 2σ(*F*^2^)] = 0.041	Hydrogen site location: inferred from neighbouring sites
*wR*(*F*^2^) = 0.106	H-atom parameters constrained
*S* = 0.88	*w* = 1/[σ^2^(*F*_o_^2^) + (0.0645*P*)^2^] where *P* = (*F*_o_^2^ + 2*F*_c_^2^)/3
3648 reflections	(Δ/σ)_max_ < 0.001
272 parameters	Δρ_max_ = 0.53 e Å^−^^3^
0 restraints	Δρ_min_ = −0.25 e Å^−^^3^

### Special details

**Table:**

Geometry. All e.s.d.'s (except the e.s.d. in the dihedral angle between two l.s. planes) are estimated using the full covariance matrix. The cell e.s.d.'s are taken into account individually in the estimation of e.s.d.'s in distances, angles and torsion angles; correlations between e.s.d.'s in cell parameters are only used when they are defined by crystal symmetry. An approximate (isotropic) treatment of cell e.s.d.'s is used for estimating e.s.d.'s involving l.s. planes.
Refinement. Refinement of *F*^2^ against ALL reflections. The weighted *R*-factor *wR* and goodness of fit *S* are based on *F*^2^, conventional *R*-factors *R* are based on *F*, with *F* set to zero for negative *F*^2^. The threshold expression of *F*^2^ > 2σ(*F*^2^) is used only for calculating *R*-factors(gt) *etc*. and is not relevant to the choice of reflections for refinement. *R*-factors based on *F*^2^ are statistically about twice as large as those based on *F*, and *R*- factors based on ALL data will be even larger.

### Fractional atomic coordinates and isotropic or equivalent isotropic displacement parameters (Å^2^)

**Table:**

	*x*	*y*	*z*	*U*_iso_*/*U*_eq_	
O1	0.70469 (14)	0.07427 (5)	0.94015 (15)	0.0323 (3)	
O2	0.63111 (15)	0.23091 (5)	0.61207 (17)	0.0406 (4)	
N1	0.57413 (16)	0.17819 (6)	0.85742 (19)	0.0350 (4)	
H1A	0.6030	0.1960	0.9464	0.042*	
N2	0.73524 (16)	0.06678 (6)	0.71431 (16)	0.0228 (4)	
C1	0.4160 (2)	0.16879 (7)	0.7732 (2)	0.0262 (5)	
C2	0.3379 (2)	0.22270 (7)	0.6974 (2)	0.0322 (5)	
H2A	0.3761	0.2355	0.6203	0.039*	
H2B	0.3585	0.2518	0.7776	0.039*	
C3	0.1719 (2)	0.21405 (8)	0.6168 (2)	0.0340 (5)	
H3A	0.1237	0.2495	0.5680	0.041*	
C4	0.1132 (2)	0.19508 (8)	0.7362 (2)	0.0390 (6)	
H4A	0.0064	0.1897	0.6853	0.047*	
H4B	0.1327	0.2239	0.8171	0.047*	
C5	0.1858 (2)	0.14072 (9)	0.8107 (2)	0.0370 (5)	
H5A	0.1469	0.1284	0.8888	0.044*	
C6	0.1570 (2)	0.09630 (8)	0.6867 (2)	0.0380 (5)	
H6A	0.2053	0.0613	0.7351	0.046*	
H6B	0.0509	0.0893	0.6353	0.046*	
C7	0.2151 (2)	0.11513 (7)	0.5674 (2)	0.0295 (5)	
H7A	0.1943	0.0861	0.4855	0.035*	
C8	0.3816 (2)	0.12404 (8)	0.6483 (2)	0.0316 (5)	
H8A	0.4292	0.0888	0.6960	0.038*	
H8B	0.4207	0.1353	0.5707	0.038*	
C9	0.3527 (2)	0.14983 (8)	0.8907 (2)	0.0350 (5)	
H9A	0.3735	0.1782	0.9729	0.042*	
H9B	0.4003	0.1148	0.9402	0.042*	
C10	0.1428 (2)	0.16958 (8)	0.4931 (2)	0.0334 (5)	
H10A	0.1818	0.1817	0.4161	0.040*	
H10B	0.0363	0.1639	0.4385	0.040*	
C11	0.6844 (2)	0.16025 (7)	0.8039 (2)	0.0265 (5)	
C12	0.70522 (19)	0.09696 (7)	0.8239 (2)	0.0256 (5)	
C13	0.72468 (19)	0.08849 (7)	0.5697 (2)	0.0234 (4)	
C14	0.74683 (19)	0.05400 (8)	0.4616 (2)	0.0271 (5)	
H14A	0.7713	0.0161	0.4860	0.033*	
C15	0.7334 (2)	0.07470 (8)	0.3187 (2)	0.0320 (5)	
H15A	0.7493	0.0508	0.2463	0.038*	
C16	0.6972 (2)	0.12964 (8)	0.2799 (2)	0.0342 (5)	
H16A	0.6870	0.1434	0.1812	0.041*	
C17	0.6760 (2)	0.16425 (8)	0.3864 (2)	0.0309 (5)	
H17A	0.6519	0.2021	0.3606	0.037*	
C18	0.68945 (19)	0.14453 (7)	0.5323 (2)	0.0250 (4)	
C19	0.6637 (2)	0.18194 (7)	0.6430 (2)	0.0285 (5)	
C20	0.8325 (2)	0.18413 (7)	0.9155 (2)	0.0344 (5)	
H20A	0.8316	0.2246	0.9049	0.052*	
H20B	0.9110	0.1685	0.8898	0.052*	
H20C	0.8488	0.1744	1.0224	0.052*	
C21	0.7661 (2)	0.00838 (7)	0.74680 (19)	0.0218 (4)	
C22	0.9084 (2)	−0.01042 (7)	0.80389 (19)	0.0255 (5)	
H22A	0.9861	0.0148	0.8194	0.031*	
C23	0.9372 (2)	−0.06604 (8)	0.8385 (2)	0.0306 (5)	
H23A	1.0347	−0.0793	0.8764	0.037*	
C24	0.8235 (2)	−0.10230 (8)	0.8176 (2)	0.0335 (5)	
H24A	0.8435	−0.1404	0.8427	0.040*	
C25	0.6813 (2)	−0.08353 (8)	0.7607 (2)	0.0350 (5)	
H25A	0.6037	−0.1087	0.7467	0.042*	
C26	0.6517 (2)	−0.02784 (7)	0.7239 (2)	0.0299 (5)	
H26A	0.5541	−0.0147	0.6834	0.036*	

### Atomic displacement parameters (Å^2^)

**Table:**

	*U*^11^	*U*^22^	*U*^33^	*U*^12^	*U*^13^	*U*^23^
O1	0.0378 (9)	0.0373 (8)	0.0255 (8)	−0.0002 (6)	0.0164 (7)	0.0002 (6)
O2	0.0436 (9)	0.0292 (8)	0.0539 (10)	0.0039 (7)	0.0245 (8)	0.0057 (7)
N1	0.0229 (10)	0.0466 (10)	0.0344 (10)	−0.0003 (8)	0.0102 (8)	−0.0187 (8)
N2	0.0263 (9)	0.0234 (8)	0.0208 (8)	0.0005 (7)	0.0117 (7)	0.0007 (7)
C1	0.0224 (12)	0.0297 (11)	0.0263 (11)	−0.0021 (8)	0.0093 (9)	−0.0035 (8)
C2	0.0351 (13)	0.0297 (11)	0.0341 (12)	−0.0013 (9)	0.0161 (10)	−0.0044 (9)
C3	0.0368 (13)	0.0327 (11)	0.0313 (12)	0.0090 (10)	0.0120 (10)	0.0020 (9)
C4	0.0324 (13)	0.0495 (13)	0.0382 (13)	−0.0009 (10)	0.0171 (11)	−0.0093 (10)
C5	0.0329 (13)	0.0547 (14)	0.0274 (12)	−0.0028 (10)	0.0161 (10)	0.0029 (10)
C6	0.0361 (13)	0.0414 (12)	0.0361 (12)	−0.0090 (10)	0.0137 (10)	0.0019 (10)
C7	0.0309 (12)	0.0328 (11)	0.0239 (11)	−0.0034 (9)	0.0097 (9)	−0.0024 (9)
C8	0.0305 (12)	0.0320 (11)	0.0326 (12)	−0.0005 (9)	0.0127 (10)	−0.0024 (9)
C9	0.0349 (13)	0.0408 (12)	0.0271 (11)	−0.0013 (10)	0.0097 (10)	−0.0006 (9)
C10	0.0328 (13)	0.0402 (12)	0.0272 (11)	−0.0022 (9)	0.0116 (10)	0.0002 (9)
C11	0.0234 (12)	0.0276 (10)	0.0294 (11)	0.0007 (8)	0.0115 (9)	−0.0020 (8)
C12	0.0205 (11)	0.0319 (11)	0.0231 (11)	0.0005 (8)	0.0074 (9)	−0.0020 (9)
C13	0.0162 (11)	0.0315 (11)	0.0225 (10)	−0.0042 (8)	0.0074 (8)	0.0000 (8)
C14	0.0248 (12)	0.0300 (11)	0.0272 (11)	−0.0018 (9)	0.0108 (9)	−0.0003 (9)
C15	0.0310 (13)	0.0428 (12)	0.0243 (11)	−0.0058 (10)	0.0130 (10)	−0.0023 (9)
C16	0.0323 (13)	0.0461 (13)	0.0250 (11)	−0.0074 (10)	0.0120 (10)	0.0060 (9)
C17	0.0247 (12)	0.0333 (11)	0.0329 (12)	−0.0048 (9)	0.0094 (10)	0.0083 (9)
C18	0.0177 (11)	0.0300 (11)	0.0277 (11)	−0.0012 (8)	0.0091 (9)	0.0038 (8)
C19	0.0192 (11)	0.0247 (11)	0.0411 (13)	−0.0003 (9)	0.0113 (10)	0.0005 (9)
C20	0.0284 (13)	0.0300 (11)	0.0423 (13)	−0.0007 (9)	0.0112 (10)	−0.0042 (9)
C21	0.0248 (12)	0.0248 (10)	0.0173 (10)	−0.0004 (9)	0.0100 (8)	−0.0015 (8)
C22	0.0260 (12)	0.0294 (11)	0.0209 (10)	−0.0038 (9)	0.0092 (9)	−0.0019 (8)
C23	0.0345 (13)	0.0319 (11)	0.0217 (11)	0.0064 (10)	0.0070 (9)	0.0010 (8)
C24	0.0531 (16)	0.0258 (11)	0.0241 (11)	0.0018 (11)	0.0177 (11)	0.0020 (9)
C25	0.0463 (15)	0.0320 (12)	0.0324 (12)	−0.0139 (10)	0.0215 (11)	−0.0052 (9)
C26	0.0256 (12)	0.0348 (12)	0.0309 (12)	−0.0028 (9)	0.0128 (9)	−0.0021 (9)

### Geometric parameters (Å, °)

**Table:**

O1—C12	1.222 (2)	C9—H9B	0.9900
O2—C19	1.229 (2)	C10—H10A	0.9900
N1—C11	1.443 (2)	C10—H10B	0.9900
N1—C1	1.478 (2)	C11—C19	1.533 (3)
N1—H1A	0.8800	C11—C12	1.541 (3)
N2—C12	1.384 (2)	C11—C20	1.548 (3)
N2—C13	1.419 (2)	C13—C14	1.394 (2)
N2—C21	1.448 (2)	C13—C18	1.405 (2)
C1—C8	1.529 (2)	C14—C15	1.387 (2)
C1—C2	1.538 (2)	C14—H14A	0.9500
C1—C9	1.539 (3)	C15—C16	1.383 (3)
C2—C3	1.540 (3)	C15—H15A	0.9500
C2—H2A	0.9900	C16—C17	1.380 (3)
C2—H2B	0.9900	C16—H16A	0.9500
C3—C10	1.521 (3)	C17—C18	1.404 (3)
C3—C4	1.523 (3)	C17—H17A	0.9500
C3—H3A	1.0000	C18—C19	1.472 (3)
C4—C5	1.527 (3)	C20—H20A	0.9800
C4—H4A	0.9900	C20—H20B	0.9800
C4—H4B	0.9900	C20—H20C	0.9800
C5—C6	1.523 (3)	C21—C22	1.381 (2)
C5—C9	1.549 (3)	C21—C26	1.384 (2)
C5—H5A	1.0000	C22—C23	1.383 (2)
C6—C7	1.517 (3)	C22—H22A	0.9500
C6—H6A	0.9900	C23—C24	1.383 (3)
C6—H6B	0.9900	C23—H23A	0.9500
C7—C10	1.528 (2)	C24—C25	1.381 (3)
C7—C8	1.546 (3)	C24—H24A	0.9500
C7—H7A	1.0000	C25—C26	1.389 (3)
C8—H8A	0.9900	C25—H25A	0.9500
C8—H8B	0.9900	C26—H26A	0.9500
C9—H9A	0.9900		
			
C11—N1—C1	124.55 (15)	C3—C10—C7	110.04 (15)
C11—N1—H1A	117.7	C3—C10—H10A	109.7
C1—N1—H1A	117.7	C7—C10—H10A	109.7
C12—N2—C13	124.03 (15)	C3—C10—H10B	109.7
C12—N2—C21	116.40 (14)	C7—C10—H10B	109.7
C13—N2—C21	119.32 (14)	H10A—C10—H10B	108.2
N1—C1—C8	112.90 (15)	N1—C11—C19	114.45 (15)
N1—C1—C2	110.97 (14)	N1—C11—C12	109.83 (15)
C8—C1—C2	108.72 (15)	C19—C11—C12	114.63 (15)
N1—C1—C9	108.31 (15)	N1—C11—C20	107.98 (15)
C8—C1—C9	107.93 (15)	C19—C11—C20	105.25 (15)
C2—C1—C9	107.85 (15)	C12—C11—C20	103.83 (14)
C1—C2—C3	111.34 (15)	O1—C12—N2	120.37 (16)
C1—C2—H2A	109.4	O1—C12—C11	120.26 (16)
C3—C2—H2A	109.4	N2—C12—C11	119.20 (16)
C1—C2—H2B	109.4	C14—C13—C18	119.17 (16)
C3—C2—H2B	109.4	C14—C13—N2	120.12 (16)
H2A—C2—H2B	108.0	C18—C13—N2	120.70 (16)
C10—C3—C4	109.61 (16)	C15—C14—C13	120.37 (17)
C10—C3—C2	108.44 (15)	C15—C14—H14A	119.8
C4—C3—C2	109.23 (16)	C13—C14—H14A	119.8
C10—C3—H3A	109.8	C16—C15—C14	120.99 (18)
C4—C3—H3A	109.8	C16—C15—H15A	119.5
C2—C3—H3A	109.8	C14—C15—H15A	119.5
C3—C4—C5	110.11 (16)	C17—C16—C15	119.10 (18)
C3—C4—H4A	109.6	C17—C16—H16A	120.5
C5—C4—H4A	109.6	C15—C16—H16A	120.5
C3—C4—H4B	109.6	C16—C17—C18	121.18 (18)
C5—C4—H4B	109.6	C16—C17—H17A	119.4
H4A—C4—H4B	108.2	C18—C17—H17A	119.4
C6—C5—C4	109.88 (16)	C17—C18—C13	119.19 (17)
C6—C5—C9	108.32 (16)	C17—C18—C19	120.23 (17)
C4—C5—C9	109.05 (16)	C13—C18—C19	120.57 (16)
C6—C5—H5A	109.9	O2—C19—C18	121.71 (18)
C4—C5—H5A	109.9	O2—C19—C11	118.74 (17)
C9—C5—H5A	109.9	C18—C19—C11	119.50 (15)
C7—C6—C5	109.93 (16)	C11—C20—H20A	109.5
C7—C6—H6A	109.7	C11—C20—H20B	109.5
C5—C6—H6A	109.7	H20A—C20—H20B	109.5
C7—C6—H6B	109.7	C11—C20—H20C	109.5
C5—C6—H6B	109.7	H20A—C20—H20C	109.5
H6A—C6—H6B	108.2	H20B—C20—H20C	109.5
C6—C7—C10	110.18 (16)	C22—C21—C26	120.75 (16)
C6—C7—C8	109.08 (16)	C22—C21—N2	120.11 (15)
C10—C7—C8	109.17 (15)	C26—C21—N2	119.11 (16)
C6—C7—H7A	109.5	C21—C22—C23	119.75 (17)
C10—C7—H7A	109.5	C21—C22—H22A	120.1
C8—C7—H7A	109.5	C23—C22—H22A	120.1
C1—C8—C7	110.40 (15)	C24—C23—C22	119.76 (19)
C1—C8—H8A	109.6	C24—C23—H23A	120.1
C7—C8—H8A	109.6	C22—C23—H23A	120.1
C1—C8—H8B	109.6	C25—C24—C23	120.53 (18)
C7—C8—H8B	109.6	C25—C24—H24A	119.7
H8A—C8—H8B	108.1	C23—C24—H24A	119.7
C1—C9—C5	111.07 (15)	C24—C25—C26	119.86 (19)
C1—C9—H9A	109.4	C24—C25—H25A	120.1
C5—C9—H9A	109.4	C26—C25—H25A	120.1
C1—C9—H9B	109.4	C21—C26—C25	119.33 (19)
C5—C9—H9B	109.4	C21—C26—H26A	120.3
H9A—C9—H9B	108.0	C25—C26—H26A	120.3
			
C11—N1—C1—C8	16.8 (2)	N1—C11—C12—N2	144.29 (16)
C11—N1—C1—C2	−105.55 (19)	C19—C11—C12—N2	13.8 (2)
C11—N1—C1—C9	136.25 (18)	C20—C11—C12—N2	−100.45 (18)
N1—C1—C2—C3	−176.90 (15)	C12—N2—C13—C14	−175.37 (16)
C8—C1—C2—C3	58.4 (2)	C21—N2—C13—C14	−1.3 (2)
C9—C1—C2—C3	−58.42 (19)	C12—N2—C13—C18	3.5 (3)
C1—C2—C3—C10	−59.6 (2)	C21—N2—C13—C18	177.59 (16)
C1—C2—C3—C4	59.8 (2)	C18—C13—C14—C15	−0.4 (3)
C10—C3—C4—C5	58.9 (2)	N2—C13—C14—C15	178.54 (16)
C2—C3—C4—C5	−59.7 (2)	C13—C14—C15—C16	−0.4 (3)
C3—C4—C5—C6	−58.9 (2)	C14—C15—C16—C17	0.8 (3)
C3—C4—C5—C9	59.7 (2)	C15—C16—C17—C18	−0.5 (3)
C4—C5—C6—C7	58.6 (2)	C16—C17—C18—C13	−0.2 (3)
C9—C5—C6—C7	−60.4 (2)	C16—C17—C18—C19	−178.86 (17)
C5—C6—C7—C10	−58.6 (2)	C14—C13—C18—C17	0.6 (3)
C5—C6—C7—C8	61.2 (2)	N2—C13—C18—C17	−178.27 (15)
N1—C1—C8—C7	178.59 (15)	C14—C13—C18—C19	179.30 (16)
C2—C1—C8—C7	−57.8 (2)	N2—C13—C18—C19	0.4 (3)
C9—C1—C8—C7	58.92 (19)	C17—C18—C19—O2	−0.5 (3)
C6—C7—C8—C1	−60.80 (19)	C13—C18—C19—O2	−179.14 (17)
C10—C7—C8—C1	59.7 (2)	C17—C18—C19—C11	−177.74 (16)
N1—C1—C9—C5	178.45 (15)	C13—C18—C19—C11	3.6 (3)
C8—C1—C9—C5	−59.0 (2)	N1—C11—C19—O2	44.2 (2)
C2—C1—C9—C5	58.3 (2)	C12—C11—C19—O2	172.40 (16)
C6—C5—C9—C1	59.9 (2)	C20—C11—C19—O2	−74.2 (2)
C4—C5—C9—C1	−59.7 (2)	N1—C11—C19—C18	−138.47 (17)
C4—C3—C10—C7	−58.7 (2)	C12—C11—C19—C18	−10.3 (2)
C2—C3—C10—C7	60.4 (2)	C20—C11—C19—C18	103.15 (18)
C6—C7—C10—C3	58.9 (2)	C12—N2—C21—C22	−101.15 (19)
C8—C7—C10—C3	−60.9 (2)	C13—N2—C21—C22	84.3 (2)
C1—N1—C11—C19	56.7 (2)	C12—N2—C21—C26	77.1 (2)
C1—N1—C11—C12	−73.9 (2)	C13—N2—C21—C26	−97.42 (19)
C1—N1—C11—C20	173.51 (16)	C26—C21—C22—C23	0.1 (3)
C13—N2—C12—O1	173.79 (16)	N2—C21—C22—C23	178.30 (15)
C21—N2—C12—O1	−0.4 (2)	C21—C22—C23—C24	−0.9 (3)
C13—N2—C12—C11	−11.0 (2)	C22—C23—C24—C25	0.8 (3)
C21—N2—C12—C11	174.78 (15)	C23—C24—C25—C26	0.0 (3)
N1—C11—C12—O1	−40.5 (2)	C22—C21—C26—C25	0.8 (3)
C19—C11—C12—O1	−170.96 (16)	N2—C21—C26—C25	−177.47 (16)
C20—C11—C12—O1	74.8 (2)	C24—C25—C26—C21	−0.8 (3)

### Hydrogen-bond geometry (Å, °)

**Table:**

*D*—H···*A*	*D*—H	H···*A*	*D*···*A*	*D*—H···*A*
N1—H1A···O2^i^	0.88	2.29	3.125 (2)	158
C25—H25A···Cg1^ii^	0.95	2.91	3.659 (2)	136

Symmetry codes: (i) *x*, −*y*+1/2, *z*+1/2; (ii) −*x*+1, −*y*, −*z*+1.
